# Deciphering the morphological, molecular, and pathogenic variability in *Fusarium* species associated with potato dry rot disease

**DOI:** 10.3389/fmicb.2025.1478798

**Published:** 2025-08-04

**Authors:** Pooja, Prashant Chauhan, Rahul Kumar Tiwari, Anil Kumar Saini, Ankit Kumar, Lellapalli Rithesh

**Affiliations:** ^1^Department of Plant Pathology, Chaudhary Charan Singh (CCS) Haryana Agricultural University, Hisar, India; ^2^Indian Institute of Sugarcane Research, Lucknow, India; ^3^Center of Food Science and Technology, Chaudhary Charan Singh Haryana Agricultural University, Hisar, India; ^4^Department of Plant Pathology, Kerala Agricultural University, Thiruvananthapuram, India

**Keywords:** dry rot, potato, morphological, molecular characterization, *Fusarium*

## Abstract

Potato (*Solanum tuberosum* L.), a member of the Solanaceae family, is a staple crop with vital importance for global food security. Various biotic and abiotic stresses affect potato crops in the field as well as in post-harvest conditions. Among biotic stresses, Fusarium dry rot, caused by the *Fusarium* species complex, is considered a major threat to potato cultivation. *Fusarium* is one of the most serious pathogens that causes dry rot in potatoes, resulting in huge yield losses. In addition, the pathogen variability depends on the ecogeographical region of potato cultivation. Therefore, to investigate the diversity, pathogenicity, and ecological variability of *Fusarium* species associated with dry rot in potatoes, 55 dry rot samples of potatoes infected with *Fusarium* spp. were collected, and from these samples, 53 *Fusarium* isolates were retrieved and characterized through micromorphological and molecular methods. The studies revealed that the isolated *Fusarium* spp. from the samples belonged to three species, namely *Fusarium sambucinum, F. oxysporum*, and *F. solani*. Among the collected species, *F. sambucinum* was the most dominant species with a high percentage of occurrence frequency. Furthermore, the pathogenicity tests of each isolate were conducted through the tuber inoculation method. The study revealed that out of all isolates, *F. sambucinum* was highly pathogenic to the susceptible potato cultivar Kufri Pukhraj. This study highlights the predominance and pathogenicity of *Fusarium sambucinum* among *Fusarium* species causing dry rot in potatoes, providing critical insights for developing targeted management strategies to mitigate yield losses and enhance potato crop resilience.

## 1 Introduction

Potato (*S. tuberosum* L.) is an important vegetable crop that belongs to the Solanaceae family and is considered a promising food source that can fulfill the food demand of the fast-growing population, which is projected to reach 10 billion by 2050 (Călinoiu et al., 2018; Levaj et al., 2023). The primary goal of potato growers is to achieve higher productivity, enhance the nutritional quality of tubers, minimize losses that may occur in the field as well as during the post-harvest period due to multiple biotic and abiotic stresses, and further implement better management practices (Tiwari et al., 2022). Various biotic and abiotic stressors include heavy rains, frost, pests, diseases, and poor post-harvest management. These stressors contribute to yield loss and deterioration of the nutritional profile in potatoes (Devaux et al., 2020). Moreover, it is reported that more than 40 pathogens and insect pests can damage both the foliage and tubers of potato plants (Tiwari et al., 2020). Several fungal diseases affect potatoes and are responsible for low tuber yield. These include late blight, black scurf, *Fusarium* wilt, powdery scab, and Fusarium dry rot (Liu et al., 2021). On the other hand, potatoes become more vulnerable to galls, blemishes, and rots owing to their higher moisture content (70%) in post-harvest storage conditions (Ranjan et al., 2021). Earlier, rot in potatoes was considered minor and inevitable. However, it has now become a major concern for potato growers. In cold storage conditions, it adversely affects seed tubers and table-purpose potatoes (Tiwari et al., 2020). Among various fungal complexes that may be responsible for rots in potatoes, *Fusarium* dry rot, caused by the *Fusarium* spp. complex, is a destructive soil-borne disease in potatoes (Falert and Akarapisan, 2019). It not only causes a yield loss of approximately 6–25% per year but also reduces the market value of potatoes worldwide (Khedher et al., 2021). The genus *Fusarium* deteriorates tuber tissues under storage conditions; however, soil- and tuber-borne inoculum can affect plants in the field (Xue et al., 2023).

*Fusarium* is one of the most destructive genera in the world as it infects almost all crop species (Christian, 2023). This genus is notable for its diversity and the range of diseases it can cause. It comprises over 300 phylogenetically distinct species, including important plant pathogens and opportunistic human pathogens. The species are categorized into several complexes, such as the *Fusarium solani* complex and the *Fusarium oxysporum* complex, which include numerous phylogenetically distinct species associated with different diseases affecting both plants and humans (Ekwomadu and Mwanza, 2023). Symptoms of dry rot include sunken and wrinkled brown–to-black tissue patches on tubers with less dry matter and shriveled flesh. During prolonged storage, the wrinkled patches produce cottony white, purple, pink, or brick orange spores and mycelial mass, which can survive in soil or decaying tuber debris (Xue and Yang, 2021). However, there is limited information available about the epidemiology of dry rot disease. The fungus can survive well at 4°C–10°C, posing an equal threat to potatoes used for processing and seed tubers (Tiwari et al., 2020). The virulence of various species of *Fusarium* depends on the cultivar types of potato and their storage conditions.

Initially, FDR was attributed to a fungus called *Fusisporium*, which was later identified as *F. oxysporum* (Tiwari et al., 2020). Currently, 17 different *Fusarium* spp. and 5 variants cause potato dry rot worldwide (Xue et al., 2023). A European project on *F. sambucinum* began in 1989, involving diverse methods and global collaboration. Among the *Fusarium* species causing potato dry rot, *F. sambucinum* is considered the most aggressive in Europe, China, and North America (Secor and Salas, 2001; Du et al., 2012). In Britain, *F. coeruleum* is the most prevalent fungus in cold storage facilities (Peters et al., 2008; Sharma et al., 2024), while *F. sulphureum* is recognized as the most frequent species across Europe and North America (Gachango et al., 2012; Zhao et al., 2024). In North Dakota, *F. graminearum* and *F. sambucinum* are the primary species associated with potato dry rot (Tiwari et al., 2020). In Michigan, *F. oxysporum* is the most common species, although *F. sambucinum* remains the most aggressive species. Interestingly, *F. graminearum*, a cereal pathogen, also plays a significant role in potato dry rot in North Dakota and Canada, likely due to wheat-potato crop rotations (Peters et al., 2008; Estrada et al., 2010; Xue et al., 2023; Daami-Remadi, 2012).

In China, *F. sambucinum* is the most aggressive species in major potato-growing regions, accompanied by *F. oxysporum*, *F. avenaceum*, *F. acuminatum*, and *F. equiseti* (Du et al., 2012; Ranjan et al., 2021). In Iran, *F. sulphureum* and *F. solani* exhibit high incidence and aggressiveness in the predominant potato cultivars. In Egypt, *F. sambucinum* is the predominant species, followed by *F. oxysporum*, *F. verticillioides*, and *F. incarnatum* (Gherbawy et al., 2019). In India, *F. sambucinum* was first reported from cold storage facilities in Madhya Pradesh, emphasizing the need for nationwide surveys to evaluate the prevalence of *Fusarium* species (Tiwari et al., 2020).

The interaction between *Fusarium* species and potato tubers is characterized by a dynamic relationship that influences disease severity and development rates (Mejdoub-Trabelsi et al., 2015; Khedher et al., 2021). The pathogen’s hemibiotrophic lifestyle, coupled with its ability to produce mycotoxins, allows it to effectively colonize and damage potato tissues (Gutiérrez-Sánchez et al., 2023). Understanding these interactions is crucial for developing effective management strategies to mitigate the impact of dry rot on potato production. In light of the growing concern regarding this disease in processing potato cultivars, the present study was conducted to survey and collect infected potato samples across various cold storage facilities and potato fields in seven major potato-growing areas in Haryana and isolate the *Fusarium* spp. responsible for potato dry rot, perform pathogenicity tests, and identify the fungal isolates through cultural, morphological, and molecular characterization.

## 2 Materials and methods

### 2.1 Survey and collection of infected potato samples

**Figure 1:**
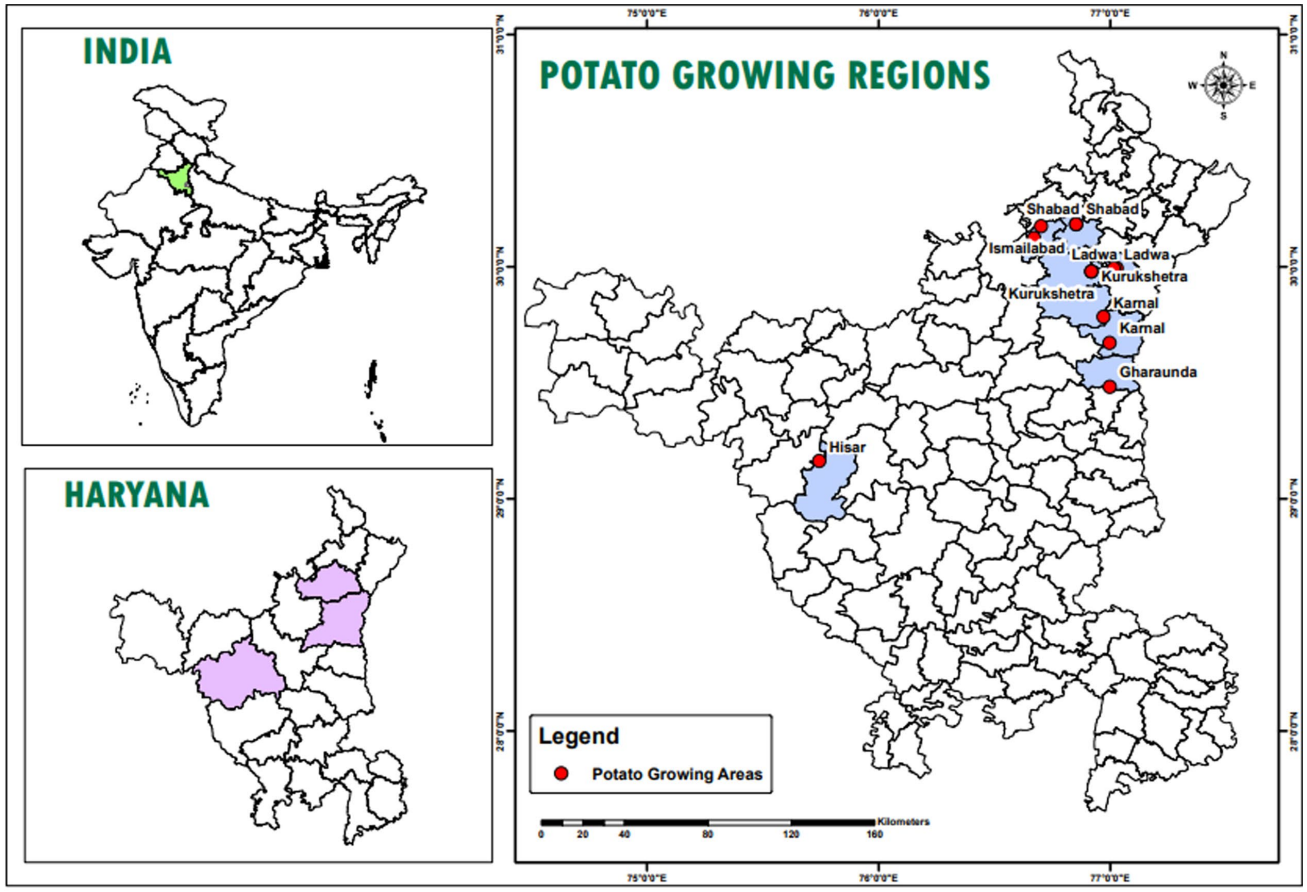
Location map of the study area which highlights different location, i.e., Shahbad, Ismailabad, Kurukshetra, Ladwa, Karnal, Gharaunda and Hisar from where dry rot infected samples collected.

**Figure 2:**
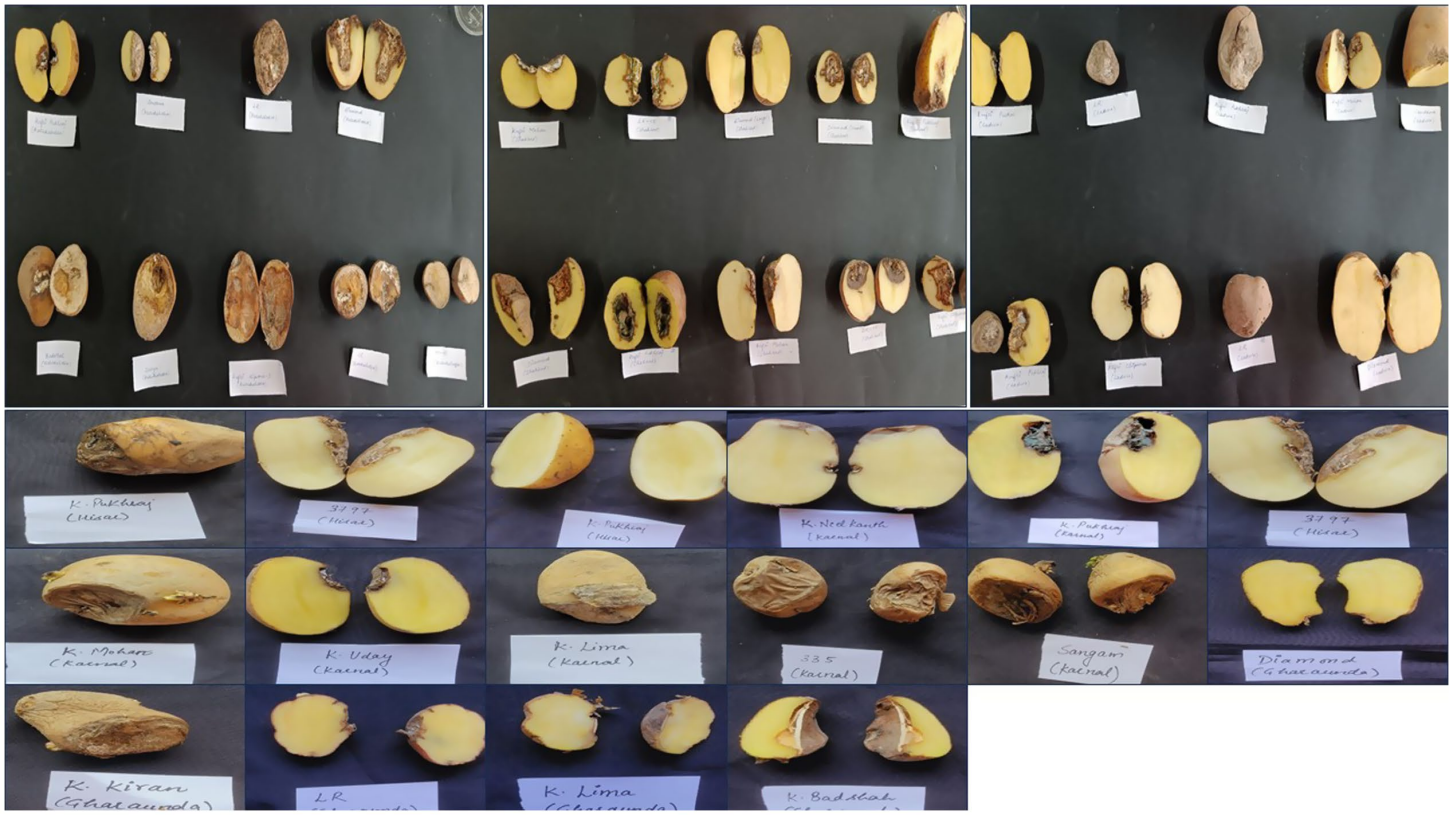
The image depicts potato tubers exhibiting dry rot symptoms, collected from different regions of Haryana.

A roving survey was conducted to collect infected samples of dry rot from cold storage facilities in the major potato-growing regions of Haryana, including Shahbad, Ismailabad, Kurukshetra, Ladwa, Karnal, Gharaunda, and Hisar (Figure 1). The study was conducted just before the sowing of potato crops in September 2023. On average, five diseased potato cultivar samples were collected from each cold storage facility based on characteristic visual symptoms. These symptoms include a pattern of brown to darker wrinkles on the skin, typically arranged in irregular concentric circles, along with the growth of whitish or pinkish fungal mycelia (Figure 2). The samples were immediately placed in a brown paper bag, brought to the plant pathology laboratory at Chaudhary Charan Singh Haryana Agricultural University (CCS HAU), Hisar, where they were stored at 4°C until further experimentation. Various isolates collected along with their identification and location details are presented in Table 1.

**Table 1:** Various isolates collected from major potato growing areas of Haryana.

**Isolate name**	**Potato variety**	**Latitude**	**Longitude**
PDR 1.1	Kufri Pukhraj	30.123232°	76.671479°
PDR 1.2	FL-54	30.123232°	76.671479°
PDR 1.3	Kufri Mohan	30.123232°	76.671479°
PDR 1.4	Kufri Chipsona-1	30.123232°	76.671479°
PDR 1.5.1	Santana	30.123232°	76.671479°
PDR 1.5.2	Santana	30.123232°	76.671479°
PDR 2.1.1	Kufri Khyati	30.174126°	76.701418°
PDR 2.1.2	Kufri Khyati	30.174126°	76.701418°
PDR 2.2	Kufri Mohan	30.174126°	76.701418°
PDR 2.2(P)	Kufri Mohan	30.174126°	76.701418°
PDR 2.3	3797	30.174126°	76.701418°
PDR 2.4	Kufri Pukhraj	30.174126°	76.701418°
PDR 2.5	Kufri Gaurav	30.174126°	76.701418°
PDR 3.2	Kufri Pukhraj	30.182576°	76.853359°
PDR 3.2.1	Kufri Pukhraj	30.182576°	76.853359°
PDR 3.3	Kufri Mohan	30.182576°	76.853359°
PDR 3.5	Kufri Chipsona	30.182576°	76.853359°
PDR 4.1	Kufri Mohan	30.182576°	76.853359°
PDR 4.2	LR-15	30.182576°	76.853359°
PDR 4.3	Diamond (Large)	30.182576°	76.853359°
PDR 4.4.1	Diamond (Small)	30.182576°	76.853359°
PDR 4.4.2	Diamond (Small)	30.182576°	76.853359°
PDR 4.5	Kufri Pukhraj	30.182576°	76.853359°
PDR 5.1	Badshah	29.978656°	76.919132°
PDR 5.2	Surya	29.978656°	76.919132°
PDR 5.3	Kufri Chipsona-3	29.978656°	76.919132°
PDR 5.4.1	LR-15	29.978656°	76.919132°
PDR 5.4.2	LR-15	29.978656°	76.919132°
PDR 6E	Kufri Pukhraj	29.978656°	76.919132°
PDR 6.1	Kufri Pukhraj	29.978656°	76.919132°
PDR 6.4	Diamond	29.978656°	76.919132°
PDR 6.5	Kufri Chipsona	29.978656°	76.919132°
PDR 6.5(P)	Kufri Chipsona	29.978656°	76.919132°
PDR 7.2	Kufri Puskar	29.992852°	77.028121°
PDR 7.4	Kufri Mohan	29.992852°	77.028121°
PDR 7.5.1	Santana	29.992852°	77.028121°
PDR 7.5.2	Santana	29.992852°	77.028121°
PDR 8.1	Kufri Puskar	29.992285°	77.016453°
PDR 8.2	Kufri Chipsona	29.992285°	77.016453°
PDR 9.1	Kufri Lima	29.162078°	75.744147°
PDR 11.1.1	3797	29.162078°	75.744147°
PDR 11.1.2	3797	29.162078°	75.744147°
PDR 11.3	Kufri Pukhraj	29.162078°	75.744147°
PDR 12.1	Kufri Lima	29.783127°	76.971122°
PDR 12.2	311	29.783127°	76.971122°
PDR 12.3	Kufri Pukhraj	29.783127°	76.971122°
PDR 12.5	Kufri Neelkanth	29.783127°	76.971122°
PDR 12.6	Kufri Uday	29.671122°	76.997699°
PDR 12.7	335	29.671122°	76.997699°
PDR 12.8	Sangam	29.671122°	76.997699°
PDR 12.9	Super-6	29.671122°	76.997699°
PDR 13.1	Kufri Badshah	29.481979°	76.998005°
PDR 13.2	Diamond	29.481979°	76.998005°
PDR 13.3	LR	29.481979°	76.998005°
PDR 13.4	Kufri Kiran	29.481979°	76.998005°

### 2.2 Isolation of pathogen

The diseased potato tubers showing characteristic symptoms of dry rot were taken for pathogen isolation. The diseased parts were washed thoroughly in distilled water to remove dust particles and surface contaminants. The tubers were then cut aseptically into small pieces and surface-sterilized with 1% sodium hypochlorite (NaOCl) solution for 30 s. Afterward, the potato pieces were taken out from the solution and washed thrice with sterilized distilled water to remove the traces of the sodium hypochlorite solution. To remove excess moisture, the cut tissues were pressed between two folds of sterilized blotting paper under aseptic conditions. Thereafter, these tissues were placed on sterilized potato sucrose agar (PSA) medium Petri plates. The plates were then incubated at 28 ± 1°C for 7 days and examined regularly to check for colony development (Yikilmazsoy and Tosun, 2021). After incubation, the resulting colonies were purified and transferred to a new PSA medium Petri plate. After proper growth of the fungi, regular subculturing was conducted to maintain a pure culture. These cultures were subcultured at 14-day interval and maintained on PSA slants at 4°C for further studies. The isolates were then subcultured at regular for further studies.

### 2.3 Cultural and morphological characterization

The isolated fungi that proved pathogenic were identified based on their cultural, morphological, and molecular characteristics. A total of 53 isolates were cultured separately on PSA medium and incubated at 28 ± 1°C for 7 days. After 7 days of incubation, cultural characteristics such as colony color, texture, and growth (mm) were recorded. The morphological characteristics such as size (μm) and shape of conidia (micro and macro) and chlamydospores were studied (Leslie and Summerell, 2006). The study was carried out using a Zeiss Axio Imager phase contrast microscope (Carl Zeiss AG, Germany), with samples mounted on slides at 400X magnification. Images were captured, and the measurement of conidia was performed using ZEN Microscopy software. All observations were recorded in triplicate for each isolate.

#### 2.3.1 Molecular characterization

The morphologically identified *Fusarium* spp. were further subjected to molecular characterization. The mycelial bits (3 mm diameter) were taken from pure cultures and grown in potato dextrose broth (PDB; Himedia Bioscience, India). Thereafter, DNA was extracted from the 3-day-old mycelium grown in PDB using a Zymo Research Fungal DNA extraction kit. The extracted DNA was diluted to 50 ng/μL in Milli-Q water and stored at −20°C for further use. Each 20 μL PCR reaction contained 10 μL of Taq buffer, 1 μL of forward and reverse primers, 2 μL of template DNA, and a final volume adjusted to 6 μL of nuclease-free water. The *Fusarium*-specific primers ITS-1 5’-TCCGTAGGTGAACCTGCGG-3′ and ITS-4 5’TCCTCCGCTTA TTGATATGC-3 were used for the amplification of genomic DNA. The amplification process involved an initial denaturation for 4 min at 95°C, 30 cycles at 95°C for 30 s, 53°C for 1 min, 72°C for 1 min, and a final extension at 72°C for 10 min, with the reaction held at 4°C afterward. The visualization of the amplified PCR product was carried out through gel electrophoresis on a 1.2% agarose gel with Tris-borate-EDTA (TBE) running buffer and stained with ethidium bromide. Gel images were captured using a gel documentation imaging system equipped with a digital camera (INTAS, Germany). The *Fusarium*-specific bands were excised from the gel, and gel extraction was performed using a Qiagen kit following the manufacturer’s protocol. The quality of the extracted DNA from the gel was assessed using a Nanodrop spectrophotometer, followed by gel electrophoresis. Then, the samples were sent for direct sequencing. The obtained sequences were analysed using BLAST, and a phylogenetic tree was constructed to show the relationships among *Fusarium* isolates based on 570 bp sequences derived from the *ITS* (Internal Transcribed Spacer) region, and the evolutionary history was inferred using the Neighbor-Joining method. The optimal tree is presented. The percentage of replicate trees in which the associated taxa clustered together in the bootstrap test (1,000 replicates) is shown next to the branches. The evolutionary distances were computed using the Maximum Composite Likelihood method and are expressed in the units of the number of base substitutions per site. All ambiguous positions were removed for each sequence pair (pairwise deletion). Phylogenetic tree analyses were conducted using MEGA11 software (Tamura et al., 2021).

### 2.4 Pathogenicity test

The pathogenicity of all *Fusarium* isolates was assessed by inoculating healthy tubers of the susceptible cultivar (Kufri Pukhraj) to verify Koch’s postulates. The healthy tubers (without visual symptoms) were washed in tap water to remove any inert materials, followed by surface sterilization with 5% sodium hypochlorite solution for 5 min. After that, the tubers were dipped in 70% ethanol for 15 s and subsequently washed with sterilized water to remove any traces of sodium hypochlorite and ethanol. Then, the tubers were wounded using a sterilized cork borer to a depth of 4 mm. The disks (4 mm diameter) were cut from 7-day-old fungal cultures grown on PSA and placed into the hole, which was subsequently sealed with the excised plug of tuber tissue to prevent saprophytic growth. Tubers were then incubated in a growth chamber for approximately 30 days at optimum temperature (18 ± 1°C) and relative humidity (80%). In the control group, a disk of pure PSA medium was used. After 2 weeks of incubation, the grown sprouts were removed from the tubers, and the fungus was re-isolated to verify Koch’s postulates from the artificially inoculated tubers. The experiment was carried out in four replicates.

Cultivar ‘Kufri Pukhraj’ was used to evaluate the susceptibility to *Fusarium* isolates. Of the 54 treatments, there were 53 *Fusarium* isolates along with a control group. A total of 4 replications per treatment were maintained during the experiment. Following the methodology of the pathogenicity test. Sampling (30 days post inoculation) was performed to evaluate the lesion diameter, lesion depth, and rot volume of the infected tubers, following the methodology established by Heltoft et al. (2015). Two perpendicular diameters of the lesion were measured, and the mean diameter (mm) was calculated for each inoculation site. Lesion depth was measured by longitudinally cutting the potato tubers at the inoculation site and measuring the depth (mm) of the lesion with the help of a scale. On the other hand, the volume of the rot was estimated using the standard formula:


$$\text{volume}=\pi r^{2}h/3$$

Where,

*r* is the radius of the lesion in mm.

*h* is the depth of the lesion in mm.

### 2.5 Statistical analysis

Data of the artificially inoculated potato samples, cultural and micromorphological parameters were analysed using a completely randomized design in SPSS software (IBM, SPSS Inc., United States). The *p*-values were calculated, and the results were expressed as CD at a significance level of 5%. The data were also analysed to determine the significance of the treatments using Tukey’s *post hoc* test (Tukey’s Multiple Range Test). The data were expressed as mean ± standard deviation, and one-way ANOVA was used for data analysis (Gomez and Gomez, 1984).

## 3 Results

### 3.1 Isolation and identification of mycoflora associated with FDR

**Figure 3:**
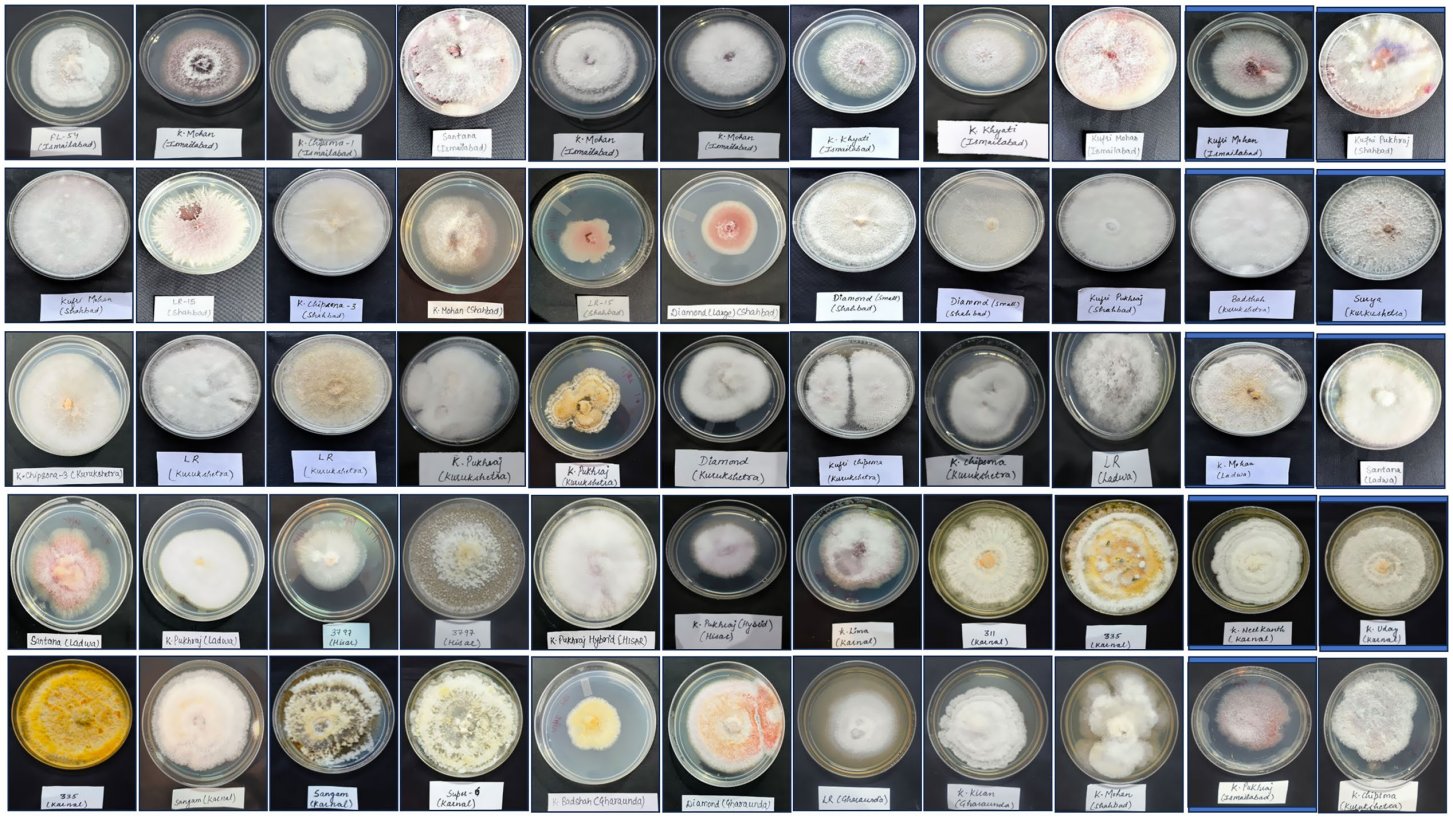
*Fusarium* isolates retrieved from infected potato tubers.

A total of 55 Fusarium isolates were retrieved on PSA medium from the infected samples by following the standard technique. Of these, 53 isolates were maintained throughout the research, while 2 were discarded due to excessive contamination in culture. The cultural and morphological variations among isolates aid in the differentiation of isolates. All isolates of *Fusarium* hold characteristic features when cultured on PSA at room temperature (28 ± 1°C). Differences in colony characters, i.e., mycelial texture, aerial mycelia, growth, pigmentation, sporulation, and colony diameter, were observed (Figure 3). The colony characteristics varied among isolates and included forms such as sparse and fluffy, cottony and dense, smooth or irregular margins, concentric ring patterns, and raised or dense growth. The isolates produced different colors, including white, pink, purple-orange-yellow, brick-orange, and violet, on the ventral surface (Table 2).

**Table 2:** Cultural characteristics of different *Fusarium* isolates retrieved from infected potato tubers.

**Isolate name**	**Cultural characters**	**Isolate name**	**Cultural characters**
**PDR 1.1**	Light violet colour with sparse white mycelia	**PDR 6E**	Brick orange mycelia with lobed margin
**PDR 1.2**	Brick orange mycelia with lobed margin	**PDR 6.1**	Brick orange mycelia with lobed margin
**PDR 1.3**	Light violet colour with sparse white mycelia	**PDR 6.4**	Brick orange mycelia with lobed margin
**PDR 1.4**	White cottony growth	**PDR 6.5**	Floccose, sparse to abundant white mycelia with a pale violet tinge
**PDR 1.5.1**	White cottony growth with violet tinge	**PDR 6.5 (P)**	Floccose, sparse to abundant white mycelia with a pale violet tinge
**PDR 1.5.2**	White cottony growth with violet tinge	**PDR 7.2**	Floccose, sparse to abundant white mycelia with a pale violet tinge
**PDR 2.1.1**	Sparse growth with light pinkish-purplish colour	**PDR 7.4**	Brick orange mycelia with lobed margin
**PDR 2.1.2**	Sparse growth with light pinkish-purplish colour	**PDR 7.5.1**	White cottony growth
**PDR 2.2(P)**	Sparse growth with purple colour	**PDR 7.5.2**	Pinkish colony with fluffy growth
**PDR 2.3**	Brick orange mycelia with lobed margin	**PDR 8.1**	Brick orange mycelia with lobed margin
**PDR 2.4**	Brick orange mycelia with lobed margin	**PDR 8.2**	Brick orange mycelia with lobed margin
**PDR 2.5**	Brick orange mycelia with lobed margin	**PDR 9.1**	Brick orange mycelia with lobed margin
**PDR 3.2**	White cottony growth	**PDR 11.1.1**	Creamish white colony
**PDR 3.2.1**	White-creamish colour mycelium	**PDR 11.1.2**	Off-white with sparse growth
**PDR 3.3**	Whitish colour with pinkish tinge	**PDR 11.3**	Floccose, sparse to abundant white mycelia with a pale violet tinge
**PDR 3.5**	Brick orange mycelia with lobed margin	**PDR 12.1**	Floccose, sparse to abundant white mycelia with a pale violet tinge
**PDR 4.1**	Sparse growth with light pinkish-purplish colour	**PDR 12.2**	Brick orange mycelia with lobed margin
**PDR 4.2**	Reddish-pink colour and sparse colony	**PDR 12.3**	Orange-whitish colour
**PDR 4.3**	Reddish-pink colour and sparse colony	**PDR 12.5**	Creamish white with ring formation
**PDR 4.4.1**	Brick orange mycelia with lobed margin	**PDR 12.6**	Brick orange mycelia with lobed margin
**PDR 4.4.2**	Brick orange mycelia with lobed margin	**PDR 12.7**	Light yellow colour
**PDR 4.5**	White cottony mycelium	**PDR 12.8**	Brick orange mycelia with lobed margin
**PDR 5.1**	White cottony mycelium	**PDR 12.9**	White colour colony
**PDR 5.2**	Sparse white colour mycelium	**PDR 13.1**	Pale yellow colour colony
**PDR 5.3**	Brick orange mycelia with lobed margin	**PDR 13.2**	Whitish-pinkish colony
**PDR 5.4.1**	White cottony mycelium with violet tinge	**PDR 13.3**	White coloured with no fluffy growth
**PDR 5.4.2**	White cottony mycelium with violet tinge	**PDR 13.4**	White fluffy growth

**Figure 4:**
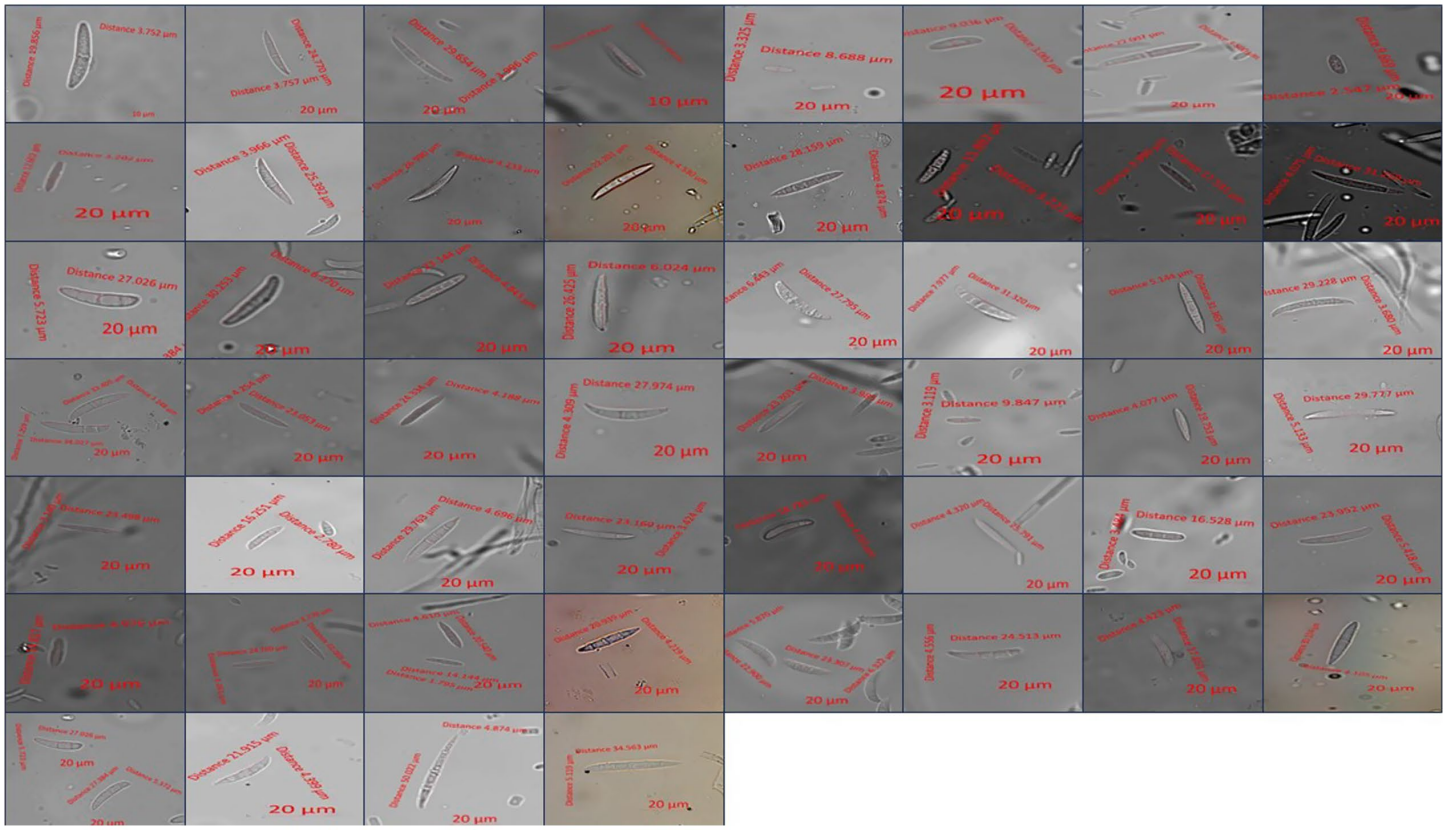
Microscopic measurement of conidia from different *Fusarium* isolates.

Micromorphological parameters, such as microconidia, macroconidia, and chlamydospores, were also recorded (Figure 4). Macroconidia (sickle-shaped to elongated with blunt ends) were abundantly generated in a 7-day-old culture, ranging from 8.69 μm to 50.02 μm × 2.55 μm to 7.98 μm in size with 3–4 septations, as shown in Table 3. The colorless microconidia were small, ovoid, and round to cylindrical, with 1–2 septa. Globose to oval, terminal, or intercalary chlamydospores with smooth or rough walls, formed singly or in chains, were observed. Based on cultural and morphological characteristics, the identified species were *F. sambucinum*, *F. solani*, and *F. oxysporum,* causing FDR in potato tubers, as per records of the *Fusarium* laboratory manual (Leslie and Summerell, 2006).

**Table 3:** Morphological characters of *Fusarium* isolates.

**Isolate name**	**Conidial width (μm)**	**Conidial length (μm)**	**Isolate name**	**Conidial width (μm)**	**Conidial length (μm)**
**PDR 1.1**	3.75±0.10^u^	19.86±0.06^wxy^	**PDR 5.4.2**	7.25±0.22^b^	33.41±1.35^b^
**PDR 1.2**	3.35±0.10^xy^	28.72±1.22^fgh^	**PDR 6.1**	4.25±0.16^opq^	23.05±0.46^qrs^
**PDR 1.3**	4.00±0.01^st^	29.67±1.23^ef^	**PDR 6.4**	4.19±0.02^qr^	24.33±0.90^nop^
**PDR 1.4**	3.49±0.14^vwx^	15.76±0.27^BC^	**PDR 6.5**	3.99±0.14^st^	23.30±0.08^pqr^
**PDR 1.5.1**	3.33±0.13^xyz^	8.69±0.05^E^	**PDR 6e**	4.31±0.04^opq^	27.97±0.50^hij^
**PDR 1.5.2**	3.00±0.01^A^	9.04±0.27^E^	**PDR 7.2**	4.08±0.04^rst^	19.74±0.11^xy^
**PDR 2.1.1**	3.50±0.07^vwx^	22.01±0.48^tuv^	**PDR 7.4**	5.17±0.06^f^	29.78±0.54^ef^
**PDR 2.1.2**	3.20±0.03^yz^	12.6±0.25^D^	**PDR 7.5.1**	3.14±0.05^zA^	23.50±0.85^opq^
**PDR 2.2**	3.96±0.06^t^	25.39±0.27^lmn^	**PDR 7.5.2**	2.78±0.07^B^	16.25±0.66^B^
**PDR 2.2 (P)**	2.55±0.09^C^	9.67±0.38^E^	**PDR 8.1**	4.70±0.11^jkl^	29.76±0.51^ef^
**PDR 2.3**	4.23±0.16^pqr^	26.99±1.14^jk^	**PDR 8.2**	4.77±0.19^jk^	34.17±1.11^b^
**PDR 2.4**	4.53±0.04^lmn^	22.20±0.30^stu^	**PDR 9.1**	4.16±0.13^qrs^	18.78±0.61^yz^
**PDR 2.5**	4.87±0.07^hij^	28.16±0.38^ghi^	**PDR 11.1.1**	4.32±0.19^opq^	18.78±0.08^yz^
**PDR 3.2**	3.22±0.03^yz^	25.56±0.82^lm^	**PDR 11.1.2**	3.48±0.10^wx^	16.53±0.21^AB^
**PDR 3.2.1**	3.34±0.10^xy^	15.89±0.47^BC^	**PDR 11.3**	5.42±0.19^e^	23.95±0.93^opq^
**PDR 3.3**	4.00±0.10^st^	17.53±0.21^A^	**PDR 12.1**	4.98±0.13^ghi^	14.83±0.13^C^
**PDR 3.5**	4.08±0.07^rst^	31.26±0.20^cd^	**PDR 12.2**	3.26±0.08^yz^	24.10±0.24^opq^
**PDR 4.1**	5.04±0.06^fgh^	30.37±0.47^cde^	**PDR 12.3**	4.61±0.17^klm^	20.54±0.13^wx^
**PDR 4.2**	3.66±0.12^uvw^	33.89±0.49^b^	**PDR 12.5**	4.22±0.11^pqr^	20.94±0.76^vw^
**PDR 4.3**	5.37±0.22^e^	27.38±0.72^ijk^	**PDR 12.6**	5.87±0.18^d^	22.90±0.02^rst^
**PDR 4.4.1**	6.27±0.13^c^	30.25±0.33^cde^	**PDR 12.7**	4.56±0.06^lmn^	24.55±0.06^mno^
**PDR 4.4.2**	4.84±0.11^ij^	23.14±0.50^qrs^	**PDR 12.8**	4.42±0.06^mno^	17.65±0.13^zA^
**PDR 4.5**	6.02±0.11^d^	26.43±0.36^kl^	**PDR 12.9**	4.11±0.06^rst^	30.17±1.17^de^
**PDR 5.1**	6.44±0.05^c^	27.80±0.85^hij^	**PDR 13.1**	5.37±0.02^e^	27.38±1.14^ijk^
**PDR 5.2**	7.98±0.14^a^	31.32±0.82^cd^	**PDR 13.2**	4.40±0.13^nop^	21.92±0.95^uv^
**PDR 5.3**	5.14±0.11^fg^	31.37±0.45^c^	**PDR 13.3**	4.87±0.21^hij^	50.02±2.12^a^
**PDR 5.4.1**	3.68±0.07^uv^	29.23±0.84^efg^	**PDR 13.4**	5.12±0.14^fg^	34.56±1.31^b^

Data are represented as mean ± standard deviation, where *n =* 3. *Different alphabets (a, b, c……) in superscripts represents significant difference (*p* < 0.05) between different isolates.

The growth rate of *Fusarium* isolates on PSA was observed periodically (24, 48, 72, 96 and 120 h), and the results indicated that 3 isolates showed a slow growth rate (≤3.0 cm colony diameter), 30 showed a medium growth rate (3.1–5.0 cm colony diameter), and 22 were fast-growing isolates (5.1–7.0 cm colony diameter) after 120 h of incubation at 28 ± 1°C (Table 4). After 120 h of incubation, isolates PDR 6.4, PDR 5.4.2, and PDR 11.1.1 showed maximum mycelial growth of 6.90, 6.40, and 6.10 cm, respectively. However, isolate PDR 1.3 showed minimum radial growth (2.40 cm).

**Table 4:** Colony growth measurement of various *Fusarium* isolates after 24, 48, 72, 96 and 120 hours.

**Isolate name**	**24 h (cm)**	**48 h (cm)**	**72 h (cm)**	**96 h (cm)**	**120 h (cm)**
**PDR 1.2**	1.25±0.03^ij^	1.45±0.06^st^	1.85±0.03^zA^	2.60±0.02^y^	3.25±0.05^r^
**PDR 1.3**	0.70±0.02^q^	0.90±0.02^x^	1.40±0.01^E^	1.90±0.02^B^	2.40±0.07^s^
**PDR 1.4**	1.05±0.02^m^	1.45±0.02^st^	2.30±0.08^tu^	3.00±0.04^vwx^	4.00±0.07^o^
**PDR 1.5.1**	1.45±0.02^f^	2.05±0.06^kl^	2.95±0.07^klm^	3.70±0.07^op^	4.90±0.01^kl^
**PDR 1.5.2**	1.30±0.03^hi^	1.85±0.04^m^	2.55±0.02^qr^	3.45±0.07^qr^	4.35±0.13^n^
**PDR 2.1.1**	1.05±0.04^m^	1.50±0.05^rs^	2.55±0.01^qr^	3.70±0.11^op^	4.65±0.07^m^
**PDR 2.1.2**	1.15±0.05^kl^	1.65±0.03^op^	2.35±0.01^stu^	3.20±0.11^tu^	4.00±0.06^o^
**PDR 2.2**	1.25±0.05^ij^	1.75±0.04^n^	2.45±0.05^rs^	3.45±0.01^qr^	4.35±0.02^n^
**PDR 2.2 (P)**	0.85±0.03^no^	1.07±0.01^w^	1.35±0.01^E^	1.95±0.06^B^	2.35±0.01^s^
**PDR 2.4**	1.35±0.01^gh^	1.90±0.06^m^	2.60±0.02^pq^	3.40±0.11^rs^	4.65±0.08^m^
**PDR 2.5**	1.55±0.06^e^	1.75±0.06^n^	2.40±0.07^st^	3.15±0.09^tuv^	4.35±0.01^n^
**PDR 3.2**	1.35±0.02^gh^	1.65±0.07^op^	2.35±0.03^stu^	2.85±0.10^x^	4.85±0.17^l^
**PDR 3.2.1**	1.20±0.04^jk^	1.60±0.03^pq^	1.95±0.03^yz^	2.45±0.06^yzA^	3.45±0.06^q^
**PDR 3.3**	1.25±0.05^ij^	2.20±0.09^i^	3.00±0.05^kl^	3.90±0.09^lmn^	5.05±0.16^jk^
**PDR 3.4**	1.45±0.01^f^	1.85±0.07^m^	2.70±0.12^op^	3.80±0.05^mno^	4.60±0.12^m^
**PDR 3.5**	0.90±0.01^n^	1.20±0.02^v^	1.55±0.01^D^	2.00±0.07^B^	2.30±0.03^s^
**PDR 4.1**	1.25±0.04^ij^	1.85±0.01^m^	3.70±0.06^e^	4.10±0.10^ijk^	5.05±0.05^jk^
**PDR 4.3**	1.37±0.02^g^	1.90±0.01^m^	2.60±0.05^pq^	3.75±0.09^nop^	5.00±0.08^jkl^
**PDR 4.4.1**	0.85±0.03^no^	1.35±0.04^u^	1.70±0.05^BC^	2.30±0.03^A^	3.70±0.01^p^
**PDR 4.4.2**	1.33±0.06^hi^	2.00±0.04^l^	2.80±0.01^no^	3.95±0.16^klm^	4.65±0.05^m^
**PDR 4.5**	0.75±0.03^pq^	1.07±0.03^w^	1.65±0.03^CD^	2.35±0.04^zA^	3.50±0.10^q^
**PDR 5.1**	1.05±0.04^m^	1.45±0.03^st^	1.81±0.03^AB^	2.51±0.01^yz^	3.20±0.11^r^
**PDR 5.2**	1.05±0.03^m^	1.55±0.02^qr^	2.10±0.05^wx^	2.95±0.01^wx^	3.70±0.01^p^
**PDR 5.3**	1.15±0.01^kl^	2.35±0.07^gh^	2.90±0.10^lmn^	3.30±0.13^rst^	4.00±0.02^o^
**PDR 5.4.1**	1.10±0.03^lm^	1.70±0.07^no^	2.40±0.03^st^	2.90±0.08^x^	3.85±0.07^op^
**PDR 5.4.2**	1.70±0.01^d^	3.00±0.01^b^	3.95±0.13^bc^	4.95±0.06^b^	6.40±0.17^b^
**PDR 6 E**	1.10±0.04^lm^	2.95±0.01^b^	2.25±0.01^uv^	3.25±0.11^stu^	4.90±0.13^kl^
**PDR 6.1**	1.10±0.03^lm^	2.35±0.01^gh^	3.35±0.02^gh^	4.15±0.16^ij^	5.00±0.18^jkl^
**PDR 6.4**	1.15±0.03^kl^	2.15±0.02^ij^	4.05±0.01^a^	5.05±0.23^a^	6.90±0.27^a^
**PDR 6.5**	0.80±0.03^op^	1.05±0.03^w^	1.90±0.06^zA^	3.10±0.02^uvw^	5.60±0.20^de^
**PDR 7.2**	1.05±0.04^m^	2.10±0.04^jk^	3.25±0.06^hi^	3.95±0.09^klm^	4.60±0.09^m^
**PDR 7.5.1**	2.30±0.01^a^	3.40±0.01^a^	4.05±0.17^a^	4.85±0.18^bc^	6.15±0.01^c^
**PDR 7.5.2**	1.35±0.03^gh^	2.30±0.09^h^	3.05±0.11^jk^	4.10±0.06^ijk^	5.00±0.13^jkl^
**PDR 8.1**	0.85±0.04^no^	1.65±0.06^op^	2.05±0.04^xy^	3.10±0.11^uvw^	4.35±0.16^n^
**PDR 8.2**	0.75±0.01^pq^	1.40±0.03^tu^	1.90±0.07^zA^	2.40±0.11^zA^	3.85±0.02^op^
**PDR 9.1**	1.15±0.05^kl^	2.00±0.01^l^	3.45±0.03^fg^	4.25±0.07^ghi^	5.05±0.20^jk^
**PDR 11.1.1**	1.30±0.01^hi^	2.55±0.02^cd^	3.75±0.08^de^	4.85±0.01^bc^	6.10±0.15^c^
**PDR 11.1.2**	1.25±0.04^ij^	2.45±0.05^ef^	3.45±0.02^fg^	4.25±0.06^ghi^	5.45±0.24^efg^
**PDR 11.2**	1.30±0.04^hi^	2.50±0.04^de^	3.05±0.04^jk^	4.10±0.05^ijk^	5.55±0.22^def^
**PDR 11.3**	1.75±0.01^d^	2.55±0.04^cd^	3.25±0.09^hi^	4.35±0.14^fgh^	5.45±0.10^efg^
**PDR 12.1**	1.35±0.05^gh^	2.55±0.08^cd^	3.85±0.14^cd^	4.85±0.09^bc^	5.35±0.01^gh^
**PDR 12.2**	1.30±0.01^hi^	2.50±0.07^de^	3.75±0.16^de^	4.70±0.07^cd^	5.65±0.20^d^
**PDR 12.3**	1.20±0.03^jk^	2.40±0.08^fg^	3.85±0.06^cd^	4.40±0.04^efg^	5.35±0.17^gh^
**PDR 12.4**	1.30±0.01^hi^	2.60±0.07^c^	3.83±0.05^d^	4.55±0.19^de^	5.40±0.04^fgh^
**PDR 12.5**	1.15±0.03^kl^	2.15±0.08^ij^	3.35±0.08^gh^	4.35±0.06^fgh^	5.50±0.04 ^def^
**PDR 12.6**	1.25±0.02^ij^	2.55±0.09^cd^	3.65±0.14^e^	4.50±0.20^ef^	5.15±0.19^ij^
**PDR 12.7**	1.70±0.03^d^	2.55±0.08^cd^	3.15±0.12^ij^	4.00±0.07^jkl^	4.65±0.20^m^
**PDR 12.8**	1.85±0.06^c^	2.60±0.09^c^	2.85±0.13^mn^	3.60±0.10^pq^	4.50±0.02^mn^
**PDR 12.9**	0.90±0.03^n^	1.55±0.07^qr^	2.17±0.04^vw^	3.30±0.01^rst^	4.50±0.09^mn^
**PDR 13.1**	2.05±0.07^b^	2.95±0.04^b^	3.30±0.10^h^	4.20±0.04^hi^	5.30±0.05^hi^
**PDR 13.2**	1.50±0.05^ef^	2.40±0.09^fg^	3.50±0.03^f^	4.15±0.14^ij^	5.00±0.05^jkl^
**PDR 13.3**	1.35±0.01^gh^	2.20±0.05^i^	3.25±0.06^hi^	3.95±0.13^klm^	4.65±0.21^m^

*Data are represented as mean ± standard deviation, where *n =* 3. *Different alphabets (a, b, c…) in superscripts represents significant difference (*p* < 0.05%) between different isolates.

### 3.2 Molecular identification of various *Fusarium* isolates based on ITS region sequencing

**Figure 5:**
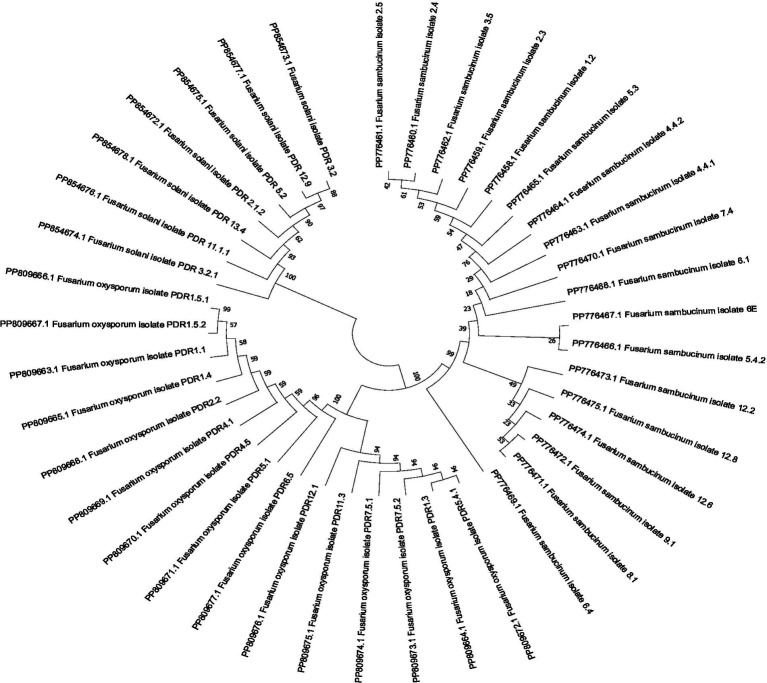
Phylogenetic tree based on internal transcribed spacer sequences.

A total of 40 isolates were selected based on cultural and morphological variability for molecular identification by using ITS primers. The remaining isolates were not identified based on molecular identification due to their morphological and cultural similarities with other isolates. ITS primer-based PCR amplification yielded a band of 570 bp, which is specific to *Fusarium* species. The bands were excised, and gel extraction was carried out followed by sequencing. Among the 40 isolates, sequencing and BLAST analysis revealed 18 isolates of *F. sambucinum*, 15 isolates of *F. oxysporum,* and 7 isolates of *F. solani*. The sequences were submitted to the GenBank database, and accession numbers were obtained (Table 5). A phylogenetic tree was constructed using 1,000 bootstrap values to check the robustness of the clades (Figure 5). For exact identification of *Fusarium* species, variations in ITS gene sequences were used in analogy with other morphological characteristics (Geiser et al., 2004). All sequences showed high similarity (more than 95%) with those of known *Fusarium* strains in GenBank.^1^ The evolutionary history was inferred using the Neighbor-Joining method. The percentage of replicate trees in which the associated taxa clustered together in the bootstrap test (1,000 replicates) is shown next to the branches. The evolutionary distances were computed using the Maximum Composite Likelihood method and are the units of the number of base substitutions per site.

**Table 5:** Molecular identification of various *Fusarium* isolates based on ITS region sequencing.

**Isolate name**	**Accession no.**	**Species**	**Isolate name**	**Accession no.**	**Species**
**PDR 1.1**	PP809663	*F. oxysporum*	**PDR 5.3**	PP776465	*F. sambucinum*
**PDR 1.2**	PP776458	*F. sambucinum*	**PDR 5.4.1**	PP809672	*F. oxysporum*
**PDR 1.3**	PP809664	*F. oxysporum*	**PDR 5.4.2**	PP776466	*F. sambucinum*
**PDR 1.4**	PP809665	*F. oxysporum*	**PDR 6E**	PP776467	*F. sambucinum*
**PDR 1.5.1**	PP809666	*F. oxysporum*	**PDR 6.1**	PP776468	*F. sambucinum*
**PDR 1.5.2**	PP809667	*F. oxysporum*	**PDR 6.4**	PP776469	*F. sambucinum*
**PDR 2.1.2**	PP854672	*F. solani*	**PDR 6.5**	PP809677	*F. oxysporum*
**PDR 2.2(P)**	PP809668	*F. oxysporum*	**PDR 7.4**	PP776470	*F. sambucinum*
**PDR 2.3**	PP776459	*F. sambucinum*	**PDR 7.5.1**	PP809674	*F. oxysporum*
**PDR 2.4**	PP776460	*F. sambucinum*	**PDR 7.5.2**	PP809673	*F. oxysporum*
**PDR 2.5**	PP776461	*F. sambucinum*	**PDR 8.1**	PP776471	*F. sambucinum*
**PDR 3.2**	PP854673	*F. solani*	**PDR 9.1**	PP776472	*F. sambucinum*
**PDR 3.2.1**	PP854674	*F. solani*	**PDR 11.1.1**	PP854676	*F. solani*
**PDR 3.5**	PP776462	*F. sambucinum*	**PDR 11.3**	PP809675	*F. oxysporum*
**PDR 4.1**	PP809669	*F. oxysporum*	**PDR 12.1**	PP809676	*F. oxysporum*
**PDR 4.4.1**	PP776463	*F. sambucinum*	**PDR 12.2**	PP776473	*F. sambucinum*
**PDR 4.4.2**	PP776464	*F. sambucinum*	**PDR 12.6**	PP776474	*F. sambucinum*
**PDR 4.5**	PP809670	*F. oxysporum*	**PDR 12.8**	PP776475	*F. sambucinum*
**PDR 5.1**	PP809671	*F. oxysporum*	**PDR 12.9**	PP854677	*F. solani*
**PDR 5.2**	PP854675	*F. solani*	**PDR 13.4**	PP854678	*F. solani*

### 3.3 Pathogenicity

**Figure 6:**
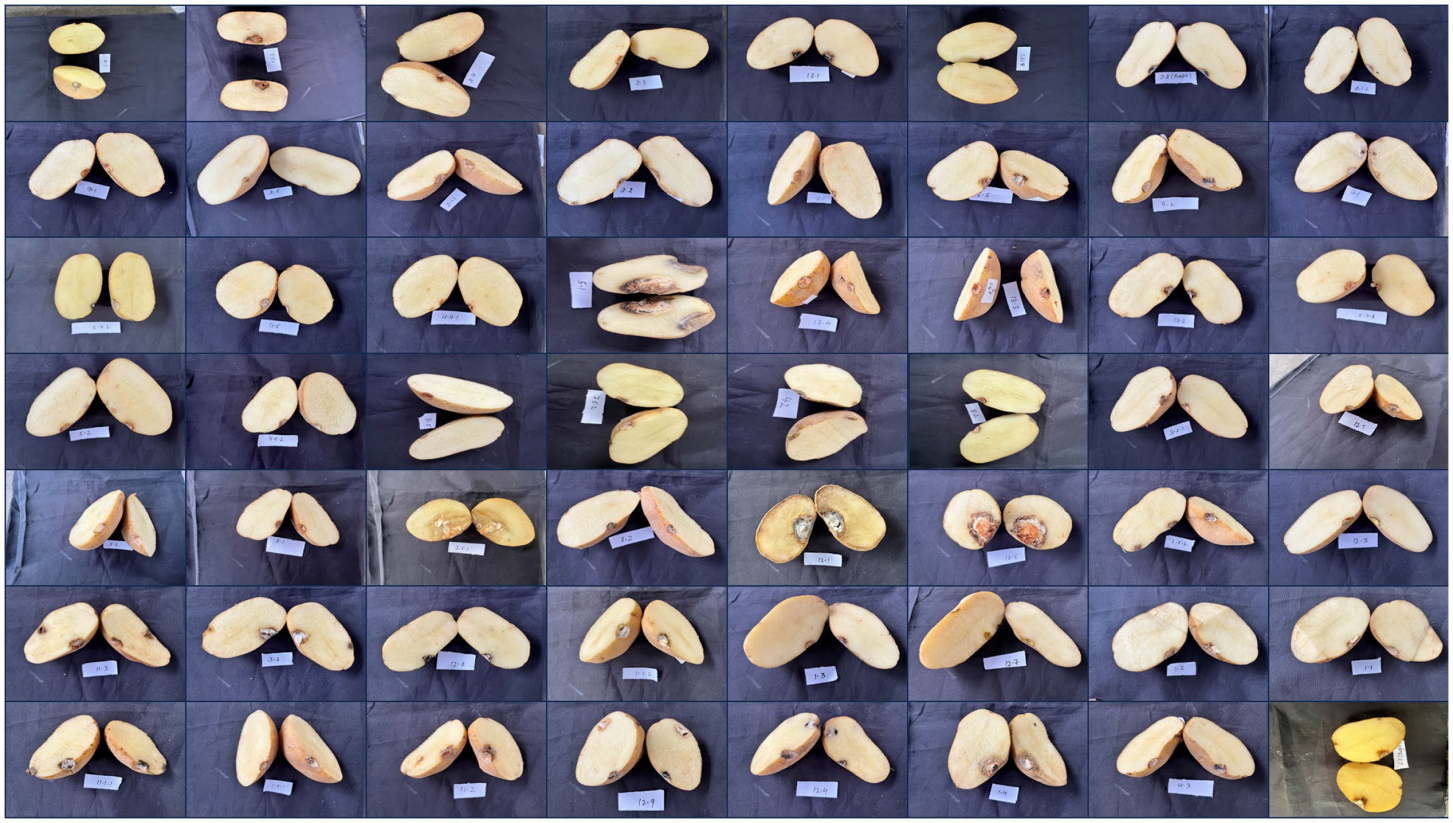
Symptomatic representation of potato tubers to confirm the pathogenicity of various *Fusarium* isolates.

The pathogenicity of the *Fusarium* isolates was confirmed under *in vitro* conditions by artificially inoculating them onto healthy potato tubers of a susceptible cultivar (Kufri Pukhraj). The initial symptoms that appeared were brown to darker wrinkles on the skin, arranged in irregular concentric circles, with whitish or pinkish fungal mycelial growth of the disease (Figure 6). Eventually, white cottony growth of the fungus, consisting of profusely branched hyphae, appeared on the rotten tissues of tubers. The control tubers (uninoculated) did not show any symptom. The symptoms were similar to FDR-infected tubers when collected. The fungal isolates were reisolated from these artificially inoculated tubers on PSA plates and exhibited the same characteristics; hence, Koch’s postulates were proved.

### 3.4 Lesion diameter, lesion depth and lesion volume

The depth, diameter, and volume of the lesions were compared between potato tubers inoculated with *Fusarium* isolates after 30 days of inoculation. The results indicate that as the disease progressed, the lesion depth, diameter, and volume increased compared to the control, as shown in Table 6. The maximum lesion diameter was observed in isolate PDR 5.1 (33 mm), followed by PDR 12.6 (32 mm), PDR 7.5.1 (26 mm), PDR 12.1 (23 mm), PDR 1.4 (17 mm), PDR 3.2, and PDR 3.3 (16 mm) (*p* < 0.05). The minimum lesion diameter was noticed in tubers inoculated with isolate PDR 2.5 (5 mm), followed by PDR 3.4 (6 mm), PDR 3.5 (6 mm), and PDR 4.1 (6 mm) (*p* < 0.05). On the other hand, the maximum lesion depth was observed in tubers inoculated with isolate PDR 7.5.1 (30 mm), followed by PDR 12.6 (29 mm), PDR 5.1 (20 mm), PDR 1.4 (15 mm), PDR 3.2 (15 mm), and PDR 3.3 (15 mm), while the minimum lesion depth was observed in tubers inoculated with isolates PDR 12.3 (3 mm) and PDR 6.1 (3 mm). The maximum lesion volume was observed in tubers inoculated with isolates PDR 12.6 (7,770 mm^3^), followed by PDR 5.1 (5,700 mm^3^) and PDR 7.5.1 (5,300 mm^3^). The isolates with minimum lesion volume were observed in tubers inoculated with PDR 2.5 (26.66 mm^3^) and PDR 6.1 (26.66 mm^3^).

**Table 6:** Effects of various *Fusarium* isolates on lesion depth, diameter and lesion volume.

**Isolate name**	**Lesion diameter (mm)**	**Lesion depth (mm)**	**Lesion volume (mm**^**3**^**)**
**PDR 1.2**	9±0.03^j^	6±0.02^k^	126.66±0.01^klm^
**PDR 1.3**	9±0.04^j^	6±0.01^k^	126.66±0.02^klm^
**PDR 1.4**	17±0.01^e^	15±0.03^d^	1133.33±0.06^e^
**PDR 1.5.1**	7±0.02^l^	5±0.02^l^	63.33±0.08^nop^
**PDR 1.5.2**	12±0.01^g^	5±0.01^l^	190.00±0.02^jk^
**PDR 2.1.1**	11±0.01^h^	6±0.02^k^	190.00±0.01^jk^
**PDR 2.1.2**	7±0.03^l^	5±0.02^l^	63.33±0.01^nop^
**PDR 2.2**	9±0.02^j^	3±0.01^n^	63.33±0.01^nop^
**PDR 2.2 (P)**	10±0.04^i^	5±0.01^l^	130.00±0.01^klm^
**PDR 2.4**	7±0.01^l^	5±0.02^l^	63.33±0.01^nop^
**PDR 2.5**	5±0.02^n^	4±0.01^m^	26.66±0.01^q^
**PDR 3.2**	16±0.01^f^	15±0.07^d^	1003.33±0.08^f^
**PDR 3.2.1**	10±0.03^i^	7±0.01^j^	183.33±0.01^jkl^
**PDR 3.3**	16±0.04^f^	15±0.01^d^	1003.33±0.10^f^
**PDR 3.4**	6±0.01^m^	7±0.02^j^	66.66±0.07^nop^
**PDR 3.5**	6±0.03^m^	5±0.01^l^	46.66±0.04^pq^
**PDR 4.1**	6±0.02^m^	5 ±0.02^l^	46.66±0.01^pq^
**PDR 4.3**	8±0.02^k^	7±0.01^j^	116.66±0.01^lmn^
**PDR 4.4.1**	7±0.01^l^	5±0.02^l^	63.33±0.02^nop^
**PDR 4.4.2**	9±0.04^j^	5±0.02^l^	106.66±0.01^mno^
**PDR 4.5**	7±0.02^l^	4±0.01^m^	50.00±0.01^opq^
**PDR 5.1**	33±0.06^a^	20±0.09^c^	5700.00±0.70^b^
**PDR 5.2**	9±0.03^j^	5±0.02^l^	106.66±0.01^mno^
**PDR 5.3**	9±0.02^j^	6±0.01^k^	126.66±0.02^klm^
**PDR 5.4.1**	10±0.01^j^	7±0.03^j^	183.33±0.20^jkl^
**PDR 5.4.2**	7±0.02^i^	4±0.01^m^	50.00±0.01^opq^
**PDR 6E**	7±0.02^l^	4 ±0.01^m^	50.00±0.01^opq^
**PDR 6.1**	6±0.02^l^	3±0.01^n^	26.66±0.01^q^
**PDR 6.4**	8±0.01^m^	4±0.01^m^	66.66±0.01^nop^
**PDR 6.5**	10±0.03^k^	12±0.03^f^	313.33±0.03^gh^
**PDR 7.2**	7±0.02^i^	5±0.02^l^	63.33±0.04^nop^
**PDR 7.5.1**	26±0.01^c^	30±0.06^a^	5306.66±0.12^c^
**PDR 7.5.2**	7±0.01^i^	5±0.01^l^	63.33±0.01^nop^
**PDR 8.1**	9±0.02^l^	5±0.01^l^	106.66±0.01^mno^
**PDR 8.2**	12±0.05^j^	10±0.03^g^	376.66±0.04^g^
**PDR 9.1**	10±0.01^g^	5±0.02^l^	130.00±0.01^klm^
**PDR 11.1.1**	12±0.02^i^	8±0.03^i^	300.00±0.04^h^
**PDR 11.1.2**	10±0.04^g^	10±0.03^g^	263.33±0.02^hi^
**PDR 11.2**	8±0.02^i^	10±0.02^g^	166.66±0.01^jkl^
**PDR 11.3**	10±0.01^k^	7±0.01^j^	183.33±0.01^jkl^
**PDR 12.1**	23±0.07^d^	14±0.02^e^	1936.66±0.03^d^
**PDR 12.2**	10±0.04^i^	12±0.03^f^	313.33±0.03^gh^
**PDR 12.3**	7±0.03^l^	3±0.01^n^	40.00±0.04^pq^
**PDR 12.4**	6±0.01^m^	6±0.01^k^	56.66±0.01^opq^
**PDR 12.5**	9±0.03^j^	5±0.01^l^	106.66±0.02^mno^
**PDR 12.6**	32±0.08^b^	29±0.13^b^	7770.00±0.60^a^
**PDR 12.7**	8±0.01^k^	5±0.02^l^	83.33±0.01^nop^
**PDR 12.8**	7±0.01^l^	5±0.01^l^	63.33±0.01^nop^
**PDR 12.9**	7±0.03^l^	4±0.01^m^	50.00±0.01^opq^
**PDR 13.1**	11±0.05^h^	9±0.02^h^	283.33±0.01^h^
**PDR 13.2**	10±0.03^i^	8±0.03^i^	210.00±0.01^ij^
**PDR 13.3**	7±0.01^l^	8±0.03^i^	103.33±0.01^mno^
**PDR 13.4**	8±0.01^k^	7±0.02^j^	116.66±0.01^lmn^
**Control**	-	-	-

*Data are represented as mean ± standard deviation, where *n =* 3. *Different alphabets (a, b, c……) in superscripts represents significant (*p* < 0.05) difference between different isolates.

## 4 Discussion

Currently, there is insufficient information on the species diversity of potato dry-rot pathogens in India. Therefore, surveys, sample collection, and pathogen identification experiments were conducted to assess the presence, distribution, and identification of causal organisms for effective plant disease management. In our study, conducted from 2023 to 2024, 55 fungal isolates were collected from various locations in Haryana and were characterized based on their cultural and morphological characteristics. Of these, 53 isolates were maintained throughout the research, while 2 were discarded due to excessive contamination in culture. Of these, 40 isolates were selected and identified at the molecular level (*ITS* sequencing). We confirmed the fungus up to species level because *Fusarium* species are widely distributed as saprophytes, soil inhabitants, and pathogens of many plants worldwide. The identification of some species is difficult and is still not clear. The fact that different species are reported under the same name and a single species under different names is also confusing (Theron, 1999). The majority of research on FDR focuses on strains of *F. solani* and *F. sambucinum*. However, strains of these species are often referred to by one or more of the synonyms by which they are known by, which complicates conclusions drawn from previous findings for non-specialists. Often, strains of these species have been incorrectly named or identified, resulting in confusion among researchers (Theron, 1999). New initiatives in the traditional area of morphological research, such as computer-aided identification, and modern techniques used by molecular biologists, such as analysis of *ITS*, translation elongation factor 1-*α*, ribosomal RNA sequences, and fingerprinting with polymerase chain reaction, have provided new insights into species relations (Theron, 1999). The results revealed that *F. sambucinum*, *F. oxysporum,* and *F. solani* were the most common pathogens causing dry rot in potato tubers in Haryana. This research provides valuable insights into the prevalent species of *Fusarium* in Haryana, which is the first evidence of its kind in the region. Similarly, Tiwari et al. (2021) identified *F. sambucinum* and *F. oxysporum* as major storage fungi causing *Fusarium* dry rot in potato tubers in the Jalandhar district, Punjab, India. Additionally, they isolated and identified *Fusarium proliferatum* as the causal pathogen of dry rot disease in the Moga district, Punjab, India, using morpho-molecular analysis. Kumar et al. (2016) conducted surveys in Punjab, India, and identified *F. culmorum*, *F. avenaceum* and *F. sambucinum* through morphological characters and species-specific primers. Erper et al. (2022) conducted a survey in Kyrgyzstan and identified *F. sambucinum* as the causal pathogen of dry rot in potatoes based on morphological and phylogenetic analysis, which was the first report of its kind in the country. Hussein et al. (2020) found 10 strains of *F. solani* causing dry rot of potatoes in the Upper Egypt region and identified them using morphological criteria and species-specific primers. Osawa et al. (2021) conducted studies in Nagasaki Prefecture, Japan, and identified *F. acuminatum*, *F. commune* and *F. oxysporum* as causal pathogens of dry rot of potatoes through morphological and DNA sequencing analyses. Similarly, Gherbawy et al. (2021) identified 187 isolates of *F. solani*, *F. keratoplasticum* and *F. falciforme* as the causal agents of dry rot in Upper Egypt, using morphological characteristics and molecular analyses. Azil et al. (2021) collected samples from 152 locations in Algeria and identified 13 species of *Fusarium* (*F. oxysporum*, *F. venenatum*, *F. redolens*, *F. tricinctum*, *F. sambucinum*, *F. cf*. *incarnatum-equiseti*, *F. nygamai*, *F. brachygibbosum*) and *Neocosmospora* (*N. solani*, *N. falciformis*) and found that *F. sambucinum* isolates were the most aggressive. The combination of rapid disease development, mycotoxin production, aggressive colonization ability, adaptability to storage conditions, and broad cultivar susceptibility makes *Fusarium sambucinum* the most aggressive dry rot pathogen of potato tubers. Understanding these factors is crucial for developing effective management strategies against this disease. A joint conclusion was drawn regarding the taxonomic position of *F. sambucinum* (Nirenberg, 1995a). These results led to the conclusion that strains of *F. sambucinum* and its synonyms should be divided into three species. These included *F. sambucinum*, *F. torulosum*, and *F. venenatum*. All three species included strains isolated from potatoes (Nirenberg, 1995b). The multi-species involvement of the pathogen exhibited its complexity in various regions. The findings of the study gave insight that the collection of *Fusarium* strains isolated from potato tubers in different regions might be useful to determine whether the proportions of fungal species in distant regions vary according to environmental conditions. In the future, it would also be interesting to investigate the specialization of the identified *Fusarium s*pecies not only in different potato varieties but also in other crops.

All strains that were analysed exhibited severe infection and caused significant damage to the tubers. However, the severity of infection varied from isolate to isolate [maximum lesion diameter was observed in isolate PDR 5.1 (33 mm) followed by PDR 12.6 (32 mm), PDR 7.5.1 (26 mm), PDR 12.1 (23 mm), PDR 1.4 (17 mm), PDR 3.2 and PDR 3.3 (16 mm)]. Characterization of the aggressiveness of the *Fusarium* isolates on potatoes revealed the pathogenicity variations. Some isolates caused sunken dry lesions and vascular discolouration on the tubers, whereas others caused marginal lesions on any of the inoculated tubers and showed minimal growth on the potato tuber. Our study corroborated with the earlier findings of Azil et al. (2021), who also confirmed the pathogenicity tests for a number of chosen *Fusarium* isolates on potato and found *F. sambucinum* as the most aggressive one. The variations in lesions might have been due to host-pathogen compatibility factors; however, the limitation of the experiment was that it did not reveal the pathological infection relationship. *Fusarium* infections in potato tubers, particularly those causing dry rot, are primarily attributed to various species within the *Fusarium* genus, notably *F. sambucinum*, *F. solani*, and *F. oxysporum* (Gavrilova et al., 2024). Understanding the genetic interactions and susceptibility mechanisms involved in these infections is crucial for developing resistant potato cultivars. The interaction between *Fusarium* species and potato tubers involves complex genetic factors, with specific susceptibility genes playing a critical role in the plant’s defense mechanisms (Soheili-Moghaddam et al., 2023). Understanding these interactions not only aids in the identification of pathogenic species but also paves the way for developing genetically resistant potato cultivars to combat dry rot and other fungal infections. Further research is necessary to explore the full potential of gene silencing and other biotechnological approaches for enhancing potato resistance to *Fusarium* infections (Ali et al., 2024). In several studies, silencing of susceptibility genes led to various defensive responses, including enhanced cell death and increased reactive oxygen species (ROS) levels at infection sites, which hindered pathogen colonization. Manipulating these susceptibility genes could be a viable strategy for breeding potato varieties with improved resistance to *Fusarium* infection (Gogoi et al., 2024). Additionally, we isolated more than one *Fusarium* species from one infected tuber, and this is due to the compatibility of the microbe-microbe interaction.

The decay of tubers was estimated by measuring the average penetration of pathogens. The extent of penetration varied depending on the cultivar-specific *Fusarium* isolates used for inoculation and the duration of storage after fungal infection. Interestingly, isolate PDR 12.6 (*F. sambucinum*) caused the most severe rot in terms of penetration and lesion volume when tested on the cultivar and stored at 18°C for up to 30 days. Bojanowski et al. (2013) reported that the depth of dry rot lesions varies depending on the type of cultivar and prevalence of *Fusarium* spp. Heltoft et al. (2015) also reported significant variation in lesion depth, diameter, and volume among potato cultivars, depending on infection by different *Fusarium* spp. Du et al. (2012) and Stefańczyk et al. (2016) reported the aggressiveness of *F. sambucinum* in terms of lesion depth or penetration on inoculated tubers. Similarly, Mejdoub-Trabelsi et al. (2015) found that combined inoculation of *F. sambucinum* with *F. oxysporum* resulted in greater penetration in tubers compared to individual inoculation. The greater aggressiveness of *F. sambucinum* compared to other *Fusarium* spp. in terms of dry rot disease incidence and severity has been well documented (Du et al., 2012; Gachango et al., 2012; Liu et al., 2021). Kuzdraliński et al. (2014) reported that foot and crown rot disease of wheat is aggravated in the presence of *F. graminearum*, *F. culmorum*, *F. poae* and *F. sporotrichioides*.

## 5 Conclusion

In this study, dry rot-infected potato samples were collected from Haryana to isolate and identify *Fusarium* spp. Pathogenicity was confirmed using Koch’s postulates. Overall, this study highlights the dominance of highly aggressive *Fusarium sambucinum* in the Haryana state. Further studies on the specialization of the identified *Fusarium* species, not only to different potato varieties but also to other crops, would explain the mechanisms involved. Additionally, determining the profiles of metabolites and their roles in the interactions with host plants would provide further insights. Because of the differences in potato cultivars and climatic conditions, diverse *Fusarium* species were isolated and identified from the FDR of potatoes in various regions of Haryana. Among them, *F. sambucinum* was considered the most predominant pathogenic fungus leading to the FDR of potatoes. Moreover, the varieties collected for the isolation of potatoes, and none of the varieties were reported resistant to dry rot. In addition, the cultural, morphological, and molecular analyses decipher the variability in *Fusarium* species; therefore, this study serves as the guidelines for future research. It would be interesting to determine the agroecological diversity among fungal isolates and their relationship with prevailing agrometeorological parameters. Additionally, this study could be insightful for researchers, scientists, potato growers and industrialists. Moreover, to the best of our knowledge, this is the first study in Haryana conditions and would prove to be a milestone work to build upon. Screening of resistant cultivars, genome editing, early detection markers, and other management options could be research areas for future research.

## Data Availability

The datasets presented in this study can be found in online repositories. The names of the repository/repositories and accession number(s) can be found in the article.

## Footnotes

^1^ http://www.ncbi.nlm.nih.gov/genbank
